# Real-time PCR quantification of the canine filaggrin orthologue in the skin of atopic and non-atopic dogs: a pilot study

**DOI:** 10.1186/1756-0500-4-554

**Published:** 2011-12-21

**Authors:** Joana Barros Roque, Caroline A O'Leary, Myat Kyaw-Tanner, David L Duffy, Michael Shipstone

**Affiliations:** 1 School of Veterinary Science, The University of Queensland, Gatton, Queensland 4343, Australia; 2 Centre for Companion Animal Health, School of Veterinary Science, The University of Queensland, St Lucia, Queensland 4069, Australia; 3 Genetic Epidemiology Laboratory, Queensland Institute of Medical Research, Herston, Queensland 4029, Australia; 4 Dermatology for Animals, Stafford Heights, Queensland 4053, Australia

## Abstract

**Background:**

Canine atopic dermatitis (AD) is a common inflammatory skin disease associated with defects in the epidermal barrier, particularly in West Highland white terriers (WHWTs). It shares many similarities with human AD, and so may be a useful animal model for this disease. Epidermal dysfunction in human AD can be caused by mutations in the gene encoding the epidermal protein filaggrin (*FLG*) and, in some atopic patients, be associated with altered *FLG *mRNA and protein expression in lesional and/or non-lesional skin. In experimental models of canine AD, mRNA expression of the orthologous canine *filaggrin *gene may be reduced in non-lesional skin compared with healthy controls. However, there is no published data on canine *filaggrin *mRNA expression in the skin of dogs with naturally-occurring AD. Hence, the aim of this pilot study was to develop a reverse transcriptase real-time PCR assay to compare *filaggrin *mRNA expression in the skin of atopic (n = 7) and non-atopic dogs (n = 5) from five breeds, including eight WHWTs.

**Findings:**

Overall, *filaggrin *mRNA expression in non-lesional atopic skin was decreased compared to non-lesional non-atopic skin (two fold change); however this difference was only statistically significant in the subgroup of WHWTs (*P *= 0.03).

**Conclusions:**

Although limited by the small sample size, these results indicate that, comparable to some cases of human AD, altered *filaggrin *mRNA expression may exist in the skin of some atopic dogs with naturally-occurring disease. Additional studies, including larger sample numbers, will be necessary to confirm this finding and to investigate whether mutations in the *filaggrin *gene exist and contribute to epidermal lesions of AD in dogs.

## Findings

Canine atopic dermatitis (AD) is a common inflammatory allergic skin disorder and results from a combination of genetic and environmental factors [1]. Changes in the structure, function and composition of the epidermis are present in atopic dogs compared with normal dogs, and may predispose to the development of the disease by enhancing percutaneous absorption and antigen presentation to the immune system [2]. Accordingly, mRNA expression studies have reported that genes encoding for proteins having epidermal barrier functions (e.g. loricrin, serine protease inhibitor kazal type-5) were either up-regulated or down-regulated in lesional and/or non-lesional skin of atopic dogs compared with normal dogs [3,4]. As AD in West Highland white terriers (WHWTs) is particularly common [5] and is usually a severe phenotype [6], a highly permeable epidermal barrier in these dogs with marked changes in genes encoding epidermal proteins may be expected to occur.

Many similarities exist between canine and human AD [7]. In humans with AD, 25-50% of cases have been reported to have defects in the epidermal protein filaggrin leading to an impaired epidermal barrier, and predisposing to development of the disease [8]. Filaggrin is produced by proteolytic cleavage of profilaggrin, a precursor located within the keratohyalin granules, during terminal differentiation of the epidermis [9]. Studies in humans have showed filaggrin has several important functions, including those of epidermal support, hydration, photoprotection and pH regulation [10]. Mutations in the human gene encoding profilaggrin (*FLG*) can result in loss of protein function, disruption of the normal epidermal barrier, and promotion of allergen penetrance [11,12]. In some humans with AD, these mutations may be associated with decreased immunostaining of the filaggrin protein in both lesional and non-lesional skin [13,14]. On the other hand, changes in *FLG *mRNA expression in human atopic skin are not so clear [13,15-17]. The only study that has analysed mRNA and protein expression simultaneously in human atopic patients determined that the decrease in expression in lesional skin was mostly caused by inflammatory sub-products (interleukins), and that the presence of mutations in the *FLG *gene did not significantly affect mRNA levels in non-lesional skin [13].

As in humans, studies using immunohistochemistry (IHC) and immunofluorescent (IF) methods report abnormalities in filaggrin protein expression in lesional and non-lesional skin of some dogs with both naturally-occurring and experimentally induced canine AD [18-20]. These changes include decreased filaggrin protein expression in non-lesional atopic skin compared with healthy dogs, and abnormalities in filaggrin protein structure in the lesional and non-lesional epidermis of some atopic dogs (e.g. altered IHC staining of protein terminal domains, altered IF staining pattern following allergen challenge). Such changes might suggest defects in filaggrin protein maturation and are consistent with 22% of affected dogs having loss-of-function mutations causing C-terminal truncation (as is reported in human AD) [18]. Further, filaggrin protein abnormalities were present in an atopic dog model prior to any allergen challenge, suggesting a possible underlying genetic cause [19].

The only pilot study investigating *filaggrin *mRNA expression in an experimental model of canine AD reported significant differences between atopic and non-atopic Beagles, with atopic animals showing less *filaggrin *mRNA expression before allergen challenge than healthy controls [20]. However, the orthologous canine *filaggrin *gene was not further investigated for mutations in these dogs. Mutations in the *filaggrin *gene, if present in dogs with AD, are likely to be breed-specific [21]. Further, in certain breeds (e.g. WHWTs) any *filaggrin *mutations could have a small effect size on the phenotype, and thus not affect mRNA expression and/or cause obvious structural/histopathological epidermal abnormalities [22]. Thus, while canine *filaggrin *mRNA and protein levels may be decreased in non-lesional skin of some dogs with AD, this finding may be breed-specific and/or not be detected in all dogs with naturally-occurring disease.

The aim of this pilot study was to develop a real-time quantitative (RT qPCR) assay for canine *filaggrin*, and to investigate whether reported variations in this gene mRNA and protein's expression in atopic dogs were reflected at the transcription level in dogs from different breeds with naturally-occurring disease. Such data will be useful in understanding the pathogenesis of this common canine disease which is also likely to be a useful animal model of human AD.

## Results

### *Filaggrin *mRNA expression in canine AD lesional, non-lesional and healthy skin

**Figure 1:**
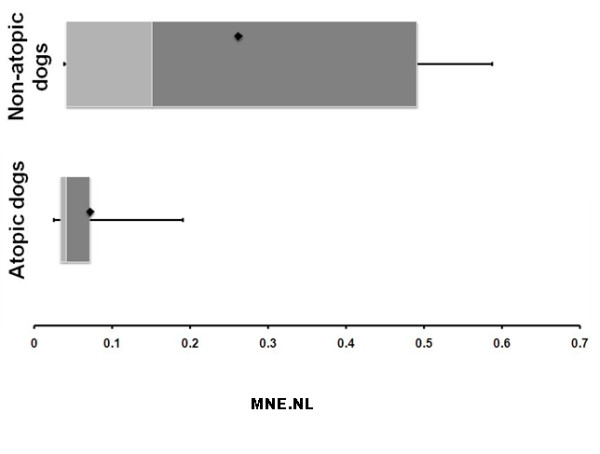
**Filaggrin mRNA expression in non-lesional skin of atopic and non-atopic dogs**. Boxplot comparing filaggrin mRNA expression (MNE.NL) in non-lesional skin of atopic and non-atopic dogs. The heavy bar is the median value, and the upper and lower limits of box are the 75th and 25th percentiles, with the bars extending to 1.5 times the interquartile range. The single square is the mean.

*Filaggrin *mRNA expression in non-lesional atopic skin (median mean normalized expression (MNE) = 0.02, range 0.01-0.04) was decreased compared with non-lesional non-atopic skin (median MNE = 0.04, range 0.01-0.16) (Figure 1), with a two-fold change in median values. However, this difference was not statistically significant (Mann-Whitney *P *= 0.34). This result did not change after removing from the analysis the two atopic dogs that had anti-inflammatory and/or immunosuppressive medication prior to the skin biopsy.

When performing this analysis in WHWTs only (n = 8), the difference in filaggrin mRNA expression between non-lesional atopic skin (median MNE = 0.04, range 0.02 to 0.07) and non-lesional non-atopic skin (median MNE = 0.4, range 0.1 to 0.6), was more pronounced (fold change of 10) and statistically significant (*P *= 0.03).

**Figure 2:**
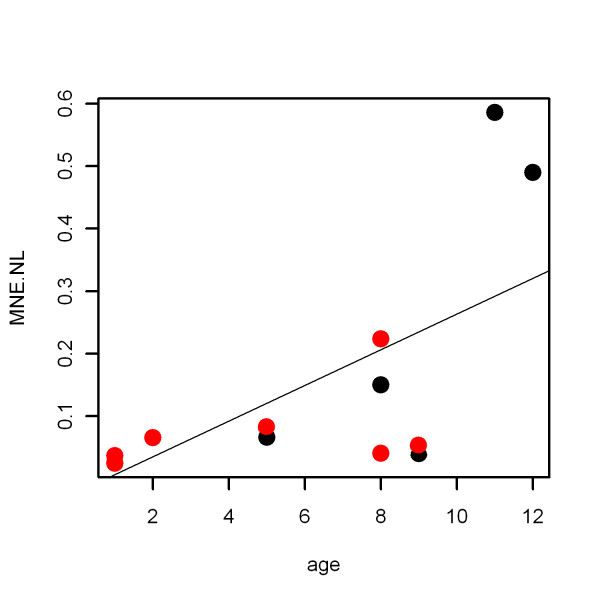
**Effect of age on filaggrin mRNA expression in non-lesional skin of atopic and non-atopic dogs**. Scatter plot showing the correlation between age of dogs (years) and filaggrin mRNA expression in non-lesional skin (MNE.NL) of atopic (red dots) and non-atopic (black dots) dogs.

The effects of age, sex and breed on MNE levels in non-lesional skin were also evaluated, since the dogs were not matched for these variables. Unlike breed and sex, age had a significant influence on MNE levels (ANOVA adjusted *P *= 0.05). A robust regression model, using iterated re-weighted least squares, was employed to attenuate the effect of extreme observations from two older dogs (Figure 2). Under this model, age was the only influential predictor (β = 0.02, t = 2.42); however it did not significantly change the estimates for the other coefficients, including clinical status (β = -0.10, t = -0.67). Hence, age did not significantly influence the difference in *filaggrin *mRNA expression between non-lesional atopic skin and non-lesional non-atopic skin.

In the one atopic WHWT dog with a lesional skin sample available, *filaggrin *mRNA expression (MNE = 1.32) was 59.8 times that measured in non-lesional skin from the same dog (MNE = 0.02), and 3.3 times the median MNE of healthy control skin.

## Discussion

The present study found that *filaggrin *mRNA expression was reduced in non-lesional atopic skin in the dogs with naturally-occurring AD compared to healthy controls (Figure 1). However, the overall difference was not statistically significant, suggesting that previous findings in atopic dog models may not be observed in all dogs with natural disease. These results are in accordance with observations in human atopic patients, where mRNA *FLG *expression is not always altered or associated with the presence of mutations and/or protein abnormalities. Alternately, variations in canine *filaggrin *mRNA levels could be specific to certain breed(s) or individual dogs with AD, and subject to phenotypic particularities [23]. Thus, in the present study, the more significant decrease in *filaggrin *mRNA expression reported in non-lesional atopic skin in WHWTs compared with atopic dogs in general could be due to variations in disease expression (e.g. differences in disease pathogenesis; particularities of the epidermal barrier) or by genetic influences (e.g. presence of filaggrin mutations) in this breed. Finally, the findings of the present study could be also attributable to the small number of available samples and/or the presence of atypical dogs in the experimental groups.

Unfortunately, only one sample from lesional atopic skin was available for study. In human AD, filaggrin protein expression can be decreased in lesional atopic skin compared with non-lesional atopic skin [13,15], but no significant difference is detected at the mRNA level [14]. In the atopic dog model, *filaggrin *mRNA expression reportedly increased with time (and with the development of skin lesions), following allergen challenge in experimentally sensitized Beagles [20]. A study using IHC to analyse filaggrin protein expression in skin from dogs of different breeds with naturally-occurring AD, reported an increased number and size of immature profilaggrin granules in one lesional hyperplastic atopic skin sample compared to non-lesional and healthy control skin [18]. Similarly, another study using IHC in dogs reported intense filaggrin protein staining in orthokeratotic hyperplastic skin disorders [24]. Therefore, the increase in *filaggrin *mRNA expression in our study could be associated with secondary epidermal changes occurring in lesional atopic skin (e.g. *filaggrin *up-regulation during inflammatory processes), or with particularities in the biopsy site (e.g. higher *filaggrin *mRNA expression in the abdominal skin). Further studies to investigate *filaggrin *mRNA expression analysis in paired lesional and non-lesional samples, including dogs with AD and other inflammatory diseases, would be required to evaluate the significance of this finding.

In this study, we also evaluated the effect of breed, age and sex on the relative expression levels of the *filaggrin *transcript. Although limited by the small number of participating dogs in this analysis, age seemed to exert the only significant influence on MNE levels, with older dogs having higher expression (Figure 2). The effect of age on filaggrin expression, if any, is not documented in dogs. However, in humans, expression level of epidermal proteins such as involucrin and keratin 17 is increased in fetal skin compared to adult skin [25]. This could indicate changes in the epidermal barrier structure and composition with age may also exist in dogs and affect filaggrin expression.

Real-Time qPCR may be a more sensitive method than IHC or IF, and so more robust to the effects of inflammation, proteolysis and other external influences [26]. Nevertheless, conclusions from RT qPCR can be limited by experimental challenges. In this particular study, one reference gene *(RPS19*) recommended as suitable for RT qPCR in atopic and healthy canine skin in a previous report [27], showed variable expression levels and suboptimal amplification efficiency during the initial optimization steps, and was thus removed from subsequent analyses.

## Conclusions

This study showed no overall significant difference in *filaggrin *mRNA expression in the non-lesional skin of atopic and non-atopic dogs with natural disease. However, significant decrease in filaggrin mRNA levels was detected when comparing non-lesional atopic skin with control skin in a subgroup of atopic WHWTs, suggesting specific changes in mRNA *filaggrin *expression may exist in AD in different breeds. Evaluation of *filaggrin *mRNA expression levels in a larger number of atopic and non-atopic animals, complemented by protein expression studies, histopathological and functional studies, and sequencing of gDNA in atopic dogs, would be useful to clarify whether mutations in *filaggrin *exist and contribute to the development of epidermal lesions in atopic dogs.

## Methods

### Animals in the study

The University of Queensland Animal and Human Ethics Committees approved this study. Seven dogs were diagnosed as having canine AD by a veterinary dermatologist, using pre-defined diagnostic criteria [28]. Five healthy dogs were selected as controls and had no history or clinical symptoms of canine AD, pyoderma, aural disease, pruritus, foot chewing or face rubbing, and no clinical conditions likely to affect the immune system.

The atopic group was composed of seven dogs (three females, mean age 4.8 years, range 1-9 years), including five WHWTs, one Beagle and one Cavalier King Charles Spaniel. The control group was composed of five dogs (four females, mean age 8.2 years, range 1-12 years), including three WHWTs, one German Shepherd and one mixed breed. In 10 of the 12 dogs enrolled in the study, 5/7 atopic and 5/5 non-atopic, any anti-inflammatory and/or immunosuppressive medication, including immunotherapy, was withdrawn at least 3 weeks prior to skin biopsy. One atopic dog had received immunotherapy less than 3 weeks prior to skin biopsy, and another atopic dog had topical corticosteroids applied twice weekly.

### Tissue collection

Six mm punch (ZebraVet QLD Pty. Ltd., Sherwood, QLD, Australia) biopsies were obtained, induced under general anaesthesia with intravenous alfaxalone (Alfaxan^®^, Jurox Pty Ltd., Rutherford, NSW, Australia) and maintained with isoflurane inhalation (I.S.O. Inhalation Anaesthetic, Veterinary Companies of Australia Pty. Ltd., Marayong, NSW, Australia). Alternately, dogs were sedated with intravenous alfaxalone and/or local prilocaine (Bomac^® ^Prilocaine Local Anaesthetic, Intervet Pty. Ltd., Bendigo, VIC, Australia) anaesthesia was used. In one dog, punch biopsy samples were obtained 5 min after euthanasia for reasons unrelated to skin disease or this study, with Pentobarbital sodium (Lethabarb^® ^Euthanasia Injection, Virbac Pty. Ltd., Crookwell, NSW, Australia). In 6/7 atopic dogs, one sample was obtained from clinically unaffected skin on the dorsal neck. In the remaining atopic dog, an extra sample was obtained from lesional skin of the right abdomen (erythematous papule of approximately 1 cm diameter). This lesion was identified as a primary lesion by the veterinary dermatologist performing the biopsy, therefore no lichenification or hyperplasia was present. In 5/5 healthy dogs one sample was collected from clinically unaffected skin on the dorsal neck. After collection, all samples were immediately immersed in RNAlater™ RNA stabilization Reagent (QIAGEN Pty. Ltd., Doncaster, VIC, Australia) and stored at -80°C.

### RNA isolation and cDNA synthesis

RNA from skin samples was isolated using Trizol reagent^® ^(Invitrogen Pty, Mulgrave, VIC, Australia), according to the manufacturer's instructions, followed by RNase-free DNase I treatment (Invitrogen Pty, Mulgrave, VIC, Australia) to remove any genomic DNA. Messenger RNA quality and concentration were analyzed spectrophotometrically (NanoDrop Technologies, Wilmington, DE, USA). One microgram of total RNA was reverse transcribed into cDNA using Reverse Transcription System (Promega, Alexandria, NSW, Australia), according to the manufacturer's instructions, and stored at -80°C until use.

### Quantitative real-time reverse transcription PCR

At the time of the study, the canine orthologue of the filaggrin gene had not been annotated in public databases. Therefore, the PCR primer set used for RT qPCR (Table 1) was designed from the conserved region of human *FLG *[GenBank:NM_002016], mouse *flg *[GenBank: J03458, AF510860] and rat *flg *[GenBank:M21759] using Primer Express software (Applied Biosystems, Foster City, CA, USA), and positioned within the last predicted exon of the canine gene. Two reference genes were selected as being most suitable in RT qPCR studies in canine skin: Ribosomal protein L8 (*RPL8*; GenBank [XM_532360]) and Ribosomal protein S19 (*RPS19*; GenBank: [XM_533657]) [27]. Primer sequences (Table 1) for these reference genes were available from a previous study [29]. The specificity of all primers was checked using Blast [30], and results were confirmed by detection of a single band of the expected product size on agarose gel electrophoresis and sequencing of PCR products. All primer sequences were synthesized by GeneWorks (Hindmarsh, SA, Australia).

**Table 1:** Primer sequences used in reverse transcriptase real-time qPCR

Gene	Forward primer sequence 5' - 3'	Reverse primer sequence 5' - 3'	Amplicon size (bp)
*filaggrin*	TCAGTTAGGATGGTGAATGTG	TCAAAAGACAAATCCAAGCT	96

*RPL8*	CCATGAATCCTGTGGAGC	GTAGAGGGTTTGCCGATG	64

*RPS19*	CCTTCCTCAAAAAGTCTGGG	GTTCTCATCGTAGGGAGCAAG	95

Amplification efficiency (E) for each gene was determined using serial a modified delta cycle threshold (Ct) method [31]; the linearity of the curve was verified from the R2 coefficient. Efficiency (E = (10 (-1/-slope) - 1) × 100%) varied between 78% (RPS19) and 110% (filaggrin) and the R2 coefficient was greater than 0.99 for all genes. GeNorm [32] and Bestkeeper [33] revealed similar expression stability for both reference genes; however RPL8 was selected for gene expression normalization due to its superior amplification efficiency (100%), and lowest coefficient of variance (6.02 ± 1.30).

The reactions were run on an ABI 7,900 HT Fast Real-Time PCR System (Applied Biosystems, Foster City, CA, USA) and consisted of equal amounts of cDNA (equivalent to 4 ng of total RNA), 5 μl of 2xSYBR Green I Master Mix (Applied Biosystem, Foster City, CA, USA) and 1 μl of forward and reverse primers (400 nm for filaggrin and RPL8 and 300 nm for RPS19). The cycling conditions were at 95°C for 10 min, followed by 45 cycles of 15 s at 95°C, and 90 s at 55°C. The specificity of the amplified products was confirmed by a melting curve analysis. Each sample was run in triplicate and results were documented as Ct, using a 0.2 detection threshold for all assays.

Data were analysed by the Sequence Detection Systems software version 2.2.2 (Applied Biosystems, Foster City, CA, USA). Mean normalized expression (MNE) and standard error (SE) were calculated as described in [34]. The mean SE between triplicates was 8.61% (range 3.30 to 14.71%).

### Statistical analysis

Data were entered in an Excel^® ^spreadsheet (Microsoft Corporation, Redmond, DC, USA). All statistical analyses were carried out within the R statistical computing environment [35]. Mann-Whitney test was used to compare *filaggrin *MNE values between non-lesional skin of atopic and non-atopic dogs. Additionally, the effect of sex, age and breed on *filaggrin *MNE values was investigated using ANOVA and regression models. The level of statistical significance was set at 0.05.

## Competing interests

The authors declare that they have no competing interests.

## Authors' contributions

JBR was responsible for all experimental procedures, analysis and interpretation of data, manuscript writing and editing; CAO conceived and coordinated the study, contributed to the experimental design and to manuscript drafting and editing; MKT contributed to the experimental design, analysis of data and to manuscript editing; DLD contributed to the experimental design, statistical analyses and manuscript editing; MS was responsible for the diagnosis and recruitment of dogs and collection of skin samples. All authors contributed to the critical revision and approved the final manuscript.
